# Comparative risk of arrhythmias associated with systemic antifungal agents: a disproportionality analysis of the FAERS database

**DOI:** 10.3389/fphar.2026.1804602

**Published:** 2026-05-26

**Authors:** Xiaohu Yang, Hao Li, Kai Liu, Lingti Kong

**Affiliations:** 1 Department of Pharmacy, The First Affiliated Hospital of Bengbu Medical University, Bengbu, China; 2 School of Pharmacy, Bengbu Medical University, Bengbu, China; 3 Institute of Emergency and Critical Care Medicine, The First Affifiliated Hospital of Bengbu Medical University, Bengbu, China; 4 State Key Laboratory of Neurology and Oncology Drug Development, Nanjing, China

**Keywords:** adverse reactions, arrhythmias, FAERS database, signal mining, systemic antifungal agents

## Abstract

**Objective:**

The real-world cardiac safety profile of systemic antifungal agents has not been thoroughly investigated. Based on the US Food and Drug Administration Adverse Event Reporting System FDA Adverse Event Reporting System database, this study analyzed the arrhythmogenic toxicity of nine systemic antifungal drugs, aiming to provide references for clinical safe medication practices.

**Research design and methods:**

Adverse events were described and classified using arrhythmogenic toxicity-related Standardized MedDRA Queries (SMQs) from the MedDRA. To identify the association between systemic antifungal agents and arrhythmogenic toxicity, this study used four algorithms: Reporting odds ratio (ROR), Proportional reporting ratio (PRR), Multi-item gamma-poisson shrinker (MGPS), and Bayesian confidence propagation Neural Network (BCPNN).

**Results:**

A total of 42,393 reports were included. The ranking of the number of positive signals across four types of SMQs was as follows: Itraconazole (4), Fluconazole (3), Posaconazole (3), Voriconazole (2), Caspofungin (1), Amphotericin B (1), Flucytosine (0), Isavuconazole (0), Micafungin (0). Itraconazole demonstrated the strongest ROR value of 2.95 in “Cardiac arrhythmia terms, nonspecific”. The highest ROR values in “Bradyarrhythmias” were 5.53 for both posaconazole and fluconazole. Fluconazole exhibited higher ROR values than other drugs in both “Tachyarrhythmias” and “Torsade de pointes/QT prolongation”, with values of 3.53 and 14.55, respectively.

**Conclusion:**

This study employed four disproportionality analysis methods to analyze the association between systemic antifungal agents and arrhythmogenic toxicity signals. Itraconazole, fluconazole, and posaconazole demonstrated stronger arrhythmogenic risks, whereas micafungin, flucytosine, and isavuconazole showed negative signals across all four SMQs. In clinical practice, individual patient risk should be comprehensively assessed to guide personalized drug selection.

## 1 Introduction

Fungal infections impose a substantial and enduring global disease burden, with over 300 million people suffering from severe fungal diseases and approximately 1.4 million deaths attributable to them annually (Denning, 2024; Kriegl et al., 2025; Zhang et al., 2023). The incidence and mortality rates associated with invasive fungal diseases continue to rise, driven by an increasing number of patients with hematological malignancies, iatrogenic or disease-related immunosuppression/immune dysfunction, and the widespread use of indwelling catheters, surgical procedures, and solid organ transplantation. The treatment of these infections remains highly dependent on systemic antifungal agents (Thompson et al., 2024).

Current clinical practice has expanded from classical azoles (e.g., fluconazole) to include newer azoles (e.g., isavuconazole) (Dagher et al., 2022; Lewis et al., 2022; Neofytos et al., 2024), echinocandins (e.g., caspofungin), polyenes (e.g., amphotericin B), and flucytosine, constituting a multi-mechanistic therapeutic arsenal. However, this broader range of drug classes has introduced more heterogeneous safety profiles (Lewis and Wiederhold, 2023; Zhang et al., 2023). Of particular concern is cardiotoxicity. Several antifungal agents can prolong the QT interval and induce Torsades de Pointes (TdP), posing a risk of sudden cardiac death. Therefore, it is necessary to clarify the arrhythmogenic toxicity risk profiles of these commonly used drugs (Cao et al., 2026; Li et al., 2023; Ohyama et al., 2023; Tan et al., 2024; Ünal Yüksekgönül et al., 2021; Yu and Liao, 2022).

Due to limitations in enrolled populations and follow-up duration, traditional randomized controlled trials have insufficient power to detect rare or delayed adverse events (AEs) in real-world settings. The US FDA Adverse Event Reporting System (FAERS), a widely used database for post-marketing drug safety surveillance, is now extensively employed for signal detection in pharmacovigilance (Buchanan and Li, 2023; Giunchi et al., 2023; Li et al., 2023; Fernandes et al., 2023; Liabeuf et al., 2025; Ohyama et al., 2023; Fusaroli et al., 2024a; Fusaroli et al., 2024b), providing evidence for label updates and risk communications (Chen et al., 2024; Giunchi et al., 2023; Li et al., 2023). Post-marketing cardiac safety evaluations of systemic antifungals using spontaneous reporting data have also expanded in recent years (Cao et al., 2026; Tan et al., 2024).

Consequently, this study utilizes FAERS as the data source to systematically analyze reports of arrhythmogenic toxicity associated with systemic antifungal agents, aiming to provide real-world evidence to inform clinical decision-making regarding their use.

## 2 Materials and methods

### 2.1 Data source

**FIGURE 1:**
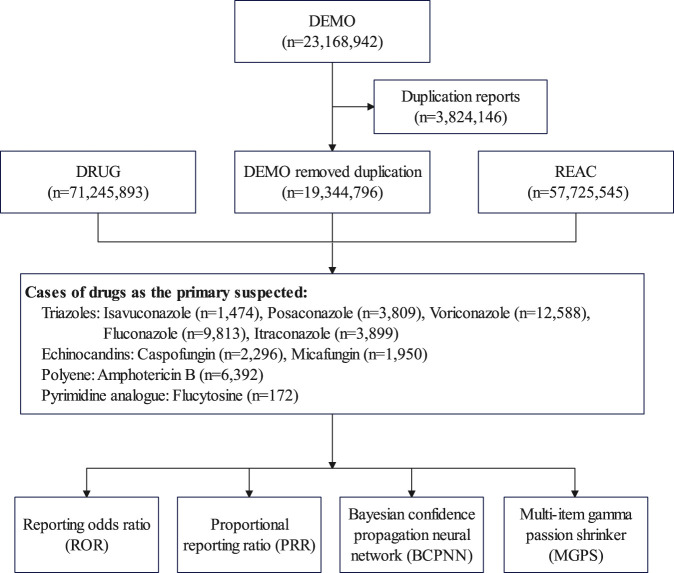
Flowchart showing data filtering.

The data for this study were sourced from the FAERS database, spanning from the first quarter of 2004 to the second quarter of 2025. The dataset comprises seven data files: patient demographic and administration information (DEMO), drug information (DRUG), adverse events (REAC), patient outcomes (OUTC), reporting source (RPSR), start and end dates for drug treatment (THE), and indications for use or diagnosis (INDI). The following sequential criteria were employed to deduplicate the dataset: (1) For identical CASEIDs, the report with the larger PRIMARYID was retained; (2) Entries with identical PRIMARYIDs were identified as duplicates and excluded. The data processing workflow was illustrated in Figure 1.

### 2.2 Data filtering

A search was conducted using the generic names (“Isavuconazole”, “Posaconazole”, “Voriconazole”, “Fluconazole”, “Itraconazole”, “Caspofungin”, “Micafungin”, “Amphotericin B”, and “Flucytosine”) and the trade names (“Cresemba”, “Noxafil”, “Vfend”, “Diflucan”, “Sporanox”, “Cancidas”, “Mycamine”, “Fungizone”, and “Ancobon”) listed as the primary suspect drugs. AEs were described and classified using arrhythmogenic toxicity-related Standardized MedDRA Queries (SMQs) from the Medical Dictionary for Regulatory Activities (MedDRA v.27.1). A total of four arrhythmia-related narrow SMQs (“Cardiac arrhythmia terms, nonspecific”, “Bradyarrhythmias”, “Tachyarrhythmias”, and “Torsade de pointes/QT prolongation, QT/TdP”) were included; the specific Preferred Terms (PTs) included in each SMQ are detailed in Supplementary Table S1 (Giunchi et al., 2023; Hung et al., 2023). We also conducted a sensitivity analysis on whether patients received chemotherapy and non-chemotherapy.

### 2.3 Data mining

To identify the association between systemic antifungal agents and arrhythmogenic toxicity, this study used four algorithms: Reporting odds ratio (ROR), Proportional reporting ratio (PRR), Multi-item gamma-poisson shrinker (MGPS), and Bayesian confidence propagation Neural Network (BCPNN). Table 1 presents the calculation formulas and positive signal thresholds for these four algorithms. To avoid false negatives, only signals that simultaneously met the positive criteria across all four algorithms were considered positive signals (Jiang et al., 2024; Li et al., 2025).

**TABLE 1:** Summary of the main algorithms used for signal detection.

Algorithm	Equation	Positive criteria
ROR	$\text{ROR}=\frac{a}{c}\times \frac{b}{d}$ $95\%\text{CI}=e^{\ln\left(\text{ROR}\right)\pm 1.96\sqrt{\frac{1}{a}+\frac{1}{b}+\frac{1}{c}+\frac{1}{d}}}$	lower limit of 95%CI>1 a≥3
PRR	$\text{PRR}=\frac{a}{\left(a+b\right)}÷\frac{c}{\left(c+d\right)}$ $\chi^{2}=\frac{{\left(\text{ad}-\text{bc}\right)}^{2}\times \left(a+b+c+d\right)}{\left(a+b\right)\left(c+d\right)\left(a+c\right)\left(d+b\right)}$	PRR≥2 $\chi^{2}\geq 4,a\geq 3$
BCPNN	$\text{IC}=\log_{2}a\left(a+b+c+d\right)\left(a+b\right)$ $\text{IC}025=e^{\ln\left(\text{IC}\right)-1.96\sqrt{\frac{1}{a}+\frac{1}{b}+\frac{1}{c}+\frac{1}{d}}}$	IC025>0
MGPS	$\text{EBGM}=\frac{a\left(a+b+c+d\right)}{\left(a+c\right)\left(a+b\right)}$ $\text{EBGM}05=e^{\ln\left(\text{EBGM}\right)-1.96\sqrt{\frac{1}{a}+\frac{1}{b}+\frac{1}{c}+\frac{1}{d}}}$	EBGM05≥2 N>0

Abbreviations: ROR: reporting odds ratio; PRR: proportional reporting ratio; BCPNN: bayesian confidence propagation neural network; MGPS: multi-item gamma passion shrinker; IC: information component; EBGM: empirical Bayes geometric mean; a: number of reports arising from the suspect adverse event (AE) and suspect drug; b: number of reports arising from the suspect AE, and all other drugs; c: number of reports arising from the suspect drug and other AEs; d: number of reports arising from other drugs and other AEs; CI: confidence interval; χ^2^: chi-squared; IC025: lower limit of 95% two-sided CI, of the IC; EBGM05: lower limit of 95% one-sided CI, of EBGM.

### 2.4 Statistical analysis

SPSS 26.0 was used for descriptive statistical analysis of the baseline characteristics of reports. R 4.2.2 software (involving the “ggplot2 3.4.2” package and the “pheatmap 1.0.12” package) was employed for disproportionality analysis and data visualization.

## 3 Results

### 3.1 Baseline characteristics

Table 2 presents the demographics of the included reports. Among reports with specified age, the age distribution was predominantly in the 18–64.9 years group, followed by the >65 years group. The types of reporters were diverse, including consumers, physicians, pharmacists, and other healthcare professionals. The reporting countries were concentrated in the United States, China, France, Japan, among others, with the United States ranking first in reports for most drugs.

**TABLE 2:** Clinical characteristics of the patients.

Characteristic	Isavuconazole	Posaconazole	Voriconazole	Fluconazole	Itraconazole	Caspofungin	Micafungin	Amphotericin B	Flucytosine
	(N = 1,474)	(N = 3,809)	(N = 12,588)	(N = 9,813)	(N = 3,899)	(N = 2,296)	(N = 1950)	(N = 6,392)	(N = 172)
Sex									
Female	570 (38.7%)	1,384 (36.3%)	4,143 (32.9%)	5,071 (51.7%)	1,511 (38.8%)	824 (35.9%)	682 (35.0%)	2,212 (34.6%)	55 (32.0%)
Male	780 (52.9%)	1904 (50.0%)	6,682 (53.1%)	3,605 (36.7%)	1,615 (41.4%)	1,192 (51.9%)	1,030 (52.8%)	3,493 (54.6%)	109 (63.4%)
Missing	124 (8.4%)	521 (13.7%)	1763 (14.0%)	1,137 (11.6%)	773 (19.8%)	280 (12.2%)	238 (12.2%)	687 (10.7%)	8 (4.7%)
Age(years)									
<18	53 (3.6%)	417 (10.9%)	1,156 (9.2%)	575 (5.9%)	286 (7.3%)	275 (12.0%)	175 (9.0%)	931 (14.6%)	5 (2.9%)
18–64.9	265 (18.0%)	1,551 (40.7%)	4,710 (37.4%)	4,439 (45.2%)	1,453 (37.3%)	1,047 (45.6%)	721 (37.0%)	3,079 (48.2%)	103 (59.9%)
>65	292 (19.8%)	900 (23.7%)	3,933 (31.2%)	2,404 (24.5%)	759 (19.5%)	549 (23.9)	335 (18.2%)	1,259 (19.7%)	51 (29.6%)
Missing	864 (58.6%)	941 (24.7%)	2,789 (22.2%)	2,395 (24.4%)	1,401 (35.9%)	425 (18.5%)	699 (35.8%)	1,123 (17.6%)	13 (7.6%)
Reporter type									
Consumer	774 (52.5%)	695 (18.2%)	2,144 (17.0%)	2,231 (22.7%)	603 (15.5%)	165 (7.2%)	980 (50.3%)	3,016 (47.2%)	4 (2.3%)
Physician	259 (17.6%)	1,178 (30.9%)	3,914 (31.1%)	3,037 (30.9%)	1,146 (29.4%)	1,119 (48.7%)	397 (20.4%)	843 (13.2%)	42 (24.4%)
Pharmacist	157 (10.7%)	378 (9.9%)	1,168 (9.3%)	929 (9.5%)	507 (13.0%)	222 (9.7%)	183 (9.4%)	471 (7.4%)	17 (9.9%)
Other health-professional	272 (18.4%)	1,503 (39.6%)	5,018 (39.9%)	3,127 (31.9%)	1,599 (41%)	733 (31.9%)	354 (18.1%)	1916 (29.9%)	105 (61.1%)
Missing	12 (0.8%)	55 (1.4%)	344 (2.7%)	489 (5.0%)	44 (1.1%)	57 (2.5%)	36 (1.8%)	146 (2.3%)	4 (2.3%)
Reporter country (TOP3)									
1	US 1131 (76.7%)	US 2051 (53.9%)	US 4698 (37.3%)	US 3701 (37.8%)	US 1016 (26.1%)	US 365 (15.9%)	US 868 (44.5%)	GB 2697 (42.2%)	US 97 (56.4%)
2	CH 126 (8.5%)	FR 385 (10.1%)	CN 1078 (8.6%)	FR 907 (9.2%)	CN 344 (8.8%)	FR 306 (13.3%)	CN 292 (15.0%)	US 1258 (19.7%)	JP 15 (8.7%)
3	JP 60 (4.1%)	CN 139 (3.6%)	FR 1020 (8.1%)	GB 870 (8.9%)	JP 294 (7.5%)	JP 261 (11.4%)	JP 242 (12.4%)	JP 252 (3.9%)	FR 10 (5.8%)

Of country abbreviation: US, united states; CH, switzerland; JP, japan; FR, france; CN, china; GB, united kiongdom.

**FIGURE 2:**
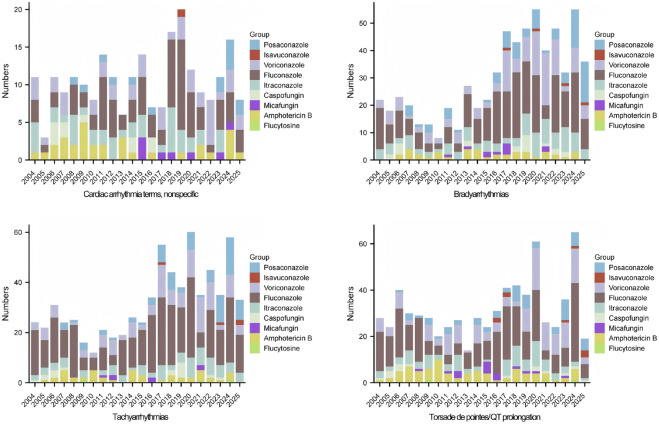
The annual trend of the number of arrhythmias related reports of each systemic antifungal agent.

The annual trend of the number of arrhythmias related reports of each drug was shown in Figure 2.

### 3.2 Signal detection

The signal strengths of systemic antifungal drugs at the SMQ level were comprehensively evaluated using four algorithms (ROR, PRR, EBGM, and IC), with the results shown in Table 3. The numbers of drug types showing positive signals in the four SMQs—“Cardiac arrhythmia terms, nonspecific”, “Bradyarrhythmias”, “Tachyarrhythmias”, and “QT/TdP”—were 1, 5, 3, and 5, respectively. Among the nine systemic antifungal drugs, only itraconazole showed positive signals in all four SMQs.

**TABLE 3:** Signal intensity of arrhythmogenic toxicity caused by systemic antifungal drugs.

SMQs	Drug	Frequency	ROR (95% CI)	PRR (χ2)	EBGM (EBGM05)	IC (IC025)	Positive signal
Cardiac arrhythmia terms, nonspecific	Isavuconazole	1	0.29 (0.04–2.07)	0.29 (1.72)	0.29 (0.04)	−1.78 (−3.19)	No
	Posaconazole	14	1.11 (0.66–1.88)	1.11 (0.17)	1.11 (0.66)	0.16 (−0.60)	No
	Voriconazole	45	0.99 (0.74–1.32)	0.99 (0.01)	0.99 (0.74)	−0.02 (−0.45)	No
	Fluconazole	83	2.13 (1.71–2.64)	2.12 (49.36)	2.12 (1.71)	1.09 (0.75)	No
	Itraconazole	43	2.95 (2.18–3.98)	2.94 (55.1)	2.94 (2.18)	1.56 (1.06)	Yes
	Caspofungin	10	1.39 (0.74–2.58)	1.38 (1.07)	1.38 (0.74)	0.47 (−0.45)	No
	Micafungin	8	1.48 (0.74–2.96)	1.48 (1.24)	1.48 (0.74)	0.56 (−0.47)	No
	Amphotericin B	32	1.59 (1.12–2.25)	1.59 (6.96)	1.59 (1.12)	0.67 (0.14)	No
	Flucytosine	0	NA	NA	NA	NA	No
Bradyarrhythmias	Isavuconazole	4	0.86 (0.32–2.28)	0.86 (0.10)	0.86 (0.32)	−0.22 (−1.47)	No
	Posaconazole	94	5.53 (4.51–6.77)	5.49 (345.15)	5.48 (4.47)	2.45 (2.09)	Yes
	Voriconazole	157	2.53 (2.16–2.96)	2.52 (144)	2.52 (2.15)	1.33 (1.09)	Yes
	Fluconazole	292	5.53 (4.92–6.20)	5.48 (1,069.16)	5.47 (4.87)	2.45 (2.26)	Yes
	Itraconazole	101	5.10 (4.19–6.20)	5.06 (329.43)	5.06 (4.16)	2.34 (2.00)	Yes
	Caspofungin	24	2.44 (1.63–3.64)	2.43 (20.32)	2.43 (1.63)	1.28 (0.62)	Yes
	Micafungin	11	1.49 (0.82–2.69)	1.49 (1.77)	1.49 (0.82)	0.57 (−0.32)	No
	Amphotericin B	45	1.64 (1.22–2.19)	1.64 (11.12)	1.63 (1.22)	0.71 (0.26)	No
	Flucytosine	0	NA	NA	NA	NA	No
Tachyarrhythmias	Isavuconazole	9	0.98 (0.51–1.88)	0.98 (0.01)	0.98 (0.51)	−0.03 (−0.95)	No
	Posaconazole	87	2.59 (2.10–3.20)	2.57 (84.01)	2.57 (2.08)	1.36 (1.03)	Yes
	Voriconazole	183	1.49 (1.29–1.72)	1.49 (29.46)	1.49 (1.29)	0.57 (0.36)	No
	Fluconazole	368	3.53 (3.19–3.91)	3.50 (658.61)	3.50 (3.15)	1.81 (1.65)	Yes
	Itraconazole	97	2.48 (2.03–3.02)	2.46 (84.65)	2.46 (2.02)	1.30 (0.99)	Yes
	Caspofungin	15	0.77 (0.46–1.28)	0.77 (1.02)	0.77 (0.46)	−0.37 (−1.08)	No
	Micafungin	20	1.37 (0.89–2.13)	1.37 (2.03)	1.37 (0.88)	0.46 (−0.2)	No
	Amphotericin B	100	1.85 (1.52–2.25)	1.84 (38.67)	1.84 (1.51)	0.88 (0.58)	No
	Flucytosine	3	2.31 (0.74–7.18)	2.30 (2.20)	2.30 (0.74)	1.20 (−0.66)	No
Torsade de pointes/QT prolongation	Isavuconazole	4	1.40 (0.53–3.74)	1.40 (0.46)	1.40 (0.53)	0.49 (−0.92)	No
	Posaconazole	102	9.85 (8.10–11.98)	9.76 (801.68)	9.75 (8.02)	3.29 (2.88)	Yes
	Voriconazole	152	4.01 (3.42–4.71)	4.00 (341.73)	3.99 (3.41)	2.00 (1.74)	Yes
	Fluconazole	464	14.55 (13.27–15.95)	14.35 (5,722.33)	14.24 (12.99)	3.83 (3.66)	Yes
	Itraconazole	90	7.44 (6.05–9.16)	7.39 (497.39)	7.38 (6.00)	2.88 (2.48)	Yes
	Caspofungin	18	3.00 (1.89–4.76)	2.99 (23.89)	2.99 (1.88)	1.58 (0.77)	No
​	Micafungin	11	2.44 (1.35–4.42)	2.44 (9.35)	2.44 (1.35)	1.29 (0.29)	No
	Amphotericin B	61	3.64 (2.83–4.69)	3.63 (116.44)	3.63 (2.82)	1.86 (1.43)	Yes
	Flucytosine	2	4.96 (1.24–19.92)	4.94 (6.30)	4.94 (1.23)	2.31 (−0.58)	No

**FIGURE 3:**
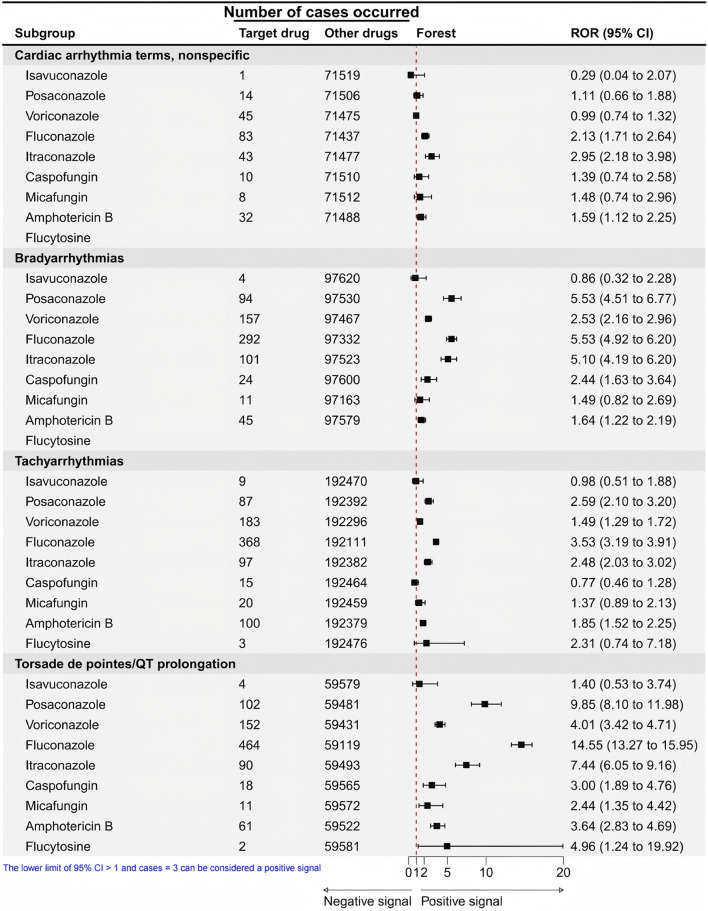
Comparison of ROR at the SMQ level for systemic antifungal agents (forest plot).

Figure 3 presents the forest plot of ROR values. Itraconazole demonstrated a ROR value of 2.95 (95% CI: 2.18–3.98) in “Cardiac arrhythmia terms, nonspecific”. The ROR values in “Bradyarrhythmias” were 5.53 for both posaconazole (95% CI: 4.51–6.77) and fluconazole (95% CI: 4.92–6.20). Fluconazole exhibited positive signal in both “Tachyarrhythmias” and “QT/TdP”, with ROR values of 3.53 (95% CI: 3.19–3.91) and 14.55 (95% CI: 13.27–15.95), respectively.

**FIGURE 4:**
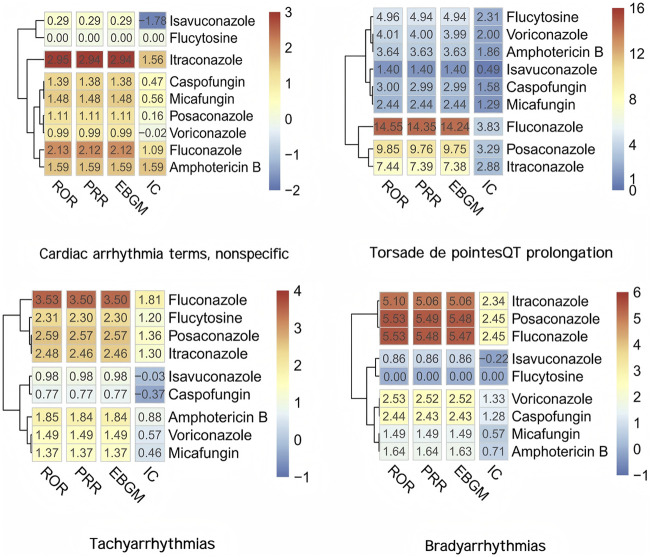
Comparison of four algorithms at the SMQ level for systemic antifungal agents (heatmap).

Figure 4 provides a more intuitive visualization of the differences among various antifungal agents across four algorithms, categorized under four SMQs.

**FIGURE 5:**
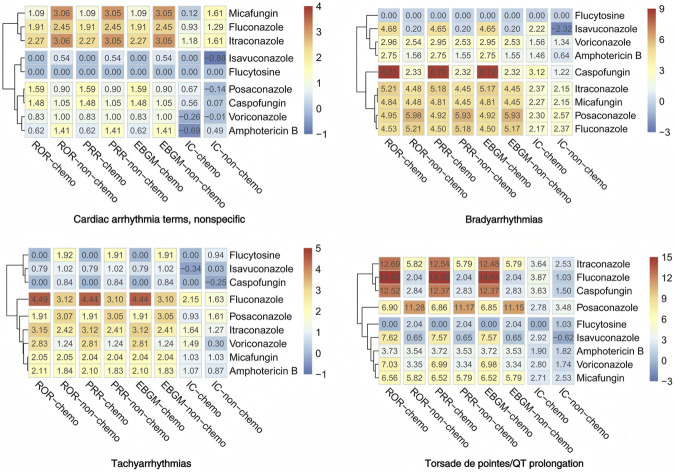
Comparison of four algorithms at the SMQ level for systemic antifungal agents in patients receiving chemotherapy and non-chemotherapy (heatmap). Abbreviations: chemo, chemotherapy; non-chemo, non-chemotherapy.

This study also conducted sensitivity analysis on whether the patients received chemotherapy and non-chemotherapy, and the results were shown in Supplementary Tables S2, S3 and Figure 5.

### 3.3 Clinical outcomes

Table 4 presents the association between the frequency of reported AEs for each drug and the clinical outcomes (including Death, Life-Threatening, Disability, Hospitalization, and Other) across different SMQs. For instance, among the adverse reactions related to fluconazole under “Cardiac arrhythmia terms, nonspecific”, the proportions resulting in Death and Life-Threatening outcomes were 16.9% and 18.1%, respectively.

**TABLE 4:** Clinical outcomes of arrhythmogenic toxicity caused by systemic antifungal drugs.

SMQ	Drug	Outcome					
		Death	Life-threatening	Disability	Hospitalization	Other	Missing
Cardiac arrhythmia terms, nonspecific	Isavuconazole	0 (0%)	1 (100%)	0 (0%)	0 (0%)	0 (0%)	0 (0%)
	Posaconazole	4 (28.6%)	5 (35.7%)	0 (0%)	1 (7.1%)	1 (7.1%)	3 (21.4%)
	Voriconazole	9 (20.5%)	7 (15.9%)	0 (0%)	2 (4.5%)	11 (25.0%)	15 (34.1%)
	Fluconazole	14 (16.9%)	15 (18.1%)	1 (1.2%)	3 (3.6%)	24 (28.9%)	26 (31.3%)
	Itraconazole	3 (7.1%)	0 (0%)	0 (0%)	7 (16.7%)	15 (35.7%)	17 (40.5%)
	Caspofungin	3 (30.0%)	0 (0%)	0 (0%)	0 (0%)	0 (0%)	7 (70.0%)
	Micafungin	4 (50.0%)	0 (0%)	0 (0%)	1 (12.5%)	3 (37.5%)	0 (0%)
	Amphotericin B	8 (25.0%)	3 (9.4%)	0 (0%)	1 (3.1%)	1 (3.1%)	19 (59.4%)
	Flucytosine	NA	NA	NA	NA	NA	NA
Bradyarrhythmias	Isavuconazole	2 (50.0%)	0 (0%)	0 (0%)	1 (25.0%)	1 (25.0%)	0 (0%)
	Posaconazole	8 (10.3%)	12 (15.4%)	0 (0%)	19 (24.4%)	26 (33.3%)	13 (16.7%)
	Voriconazole	17 (12.7%)	26 (19.4%)	0 (0%)	32 (23.9%)	30 (22.4%)	29 (21.6%)
	Fluconazole	12 (4.6%)	70 (26.6%)	0 (0%)	47 (17.9%)	75 (28.5%)	57 (21.7%)
	Itraconazole	6 (6.1%)	12 (12.2%)	0 (0%)	12 (12.2%)	41 (41.8%)	27 (27.6%)
	Caspofungin	1 (4.8%)	3 (14.3%)	0 (0%)	6 (28.6%)	5 (23.8%)	6 (28.6%)
	Micafungin	0 (0%)	0 (0%)	0 (0%)	1 (9.1%)	9 (81.8%)	1 (9.1%)
	Amphotericin B	7 (16.7%)	6 (14.3%)	0 (0%)	7 (16.7%)	9 (21.4%)	13 (31.0%)
	Flucytosine	NA	NA	NA	NA	NA	NA
Tachyarrhythmias	Isavuconazole	2 (22.2%)	1 (11.1%)	0 (0%)	2 (22.2%)	4 (44.4%)	0 (0%)
	Posaconazole	7 (11.1%)	21 (33.3%)	0 (0%)	15 (23.8%)	6 (9.5%)	14 (22.2%)
	Voriconazole	28 (19.0%)	25 (17.0%)	0 (0%)	25 (17.0%)	26 (17.7%)	43 (29.3%)
	Fluconazole	11 (4.0%)	80 (28.9%)	1 (0.4%)	41 (14.8%)	44 (15.9%)	100 (36.1%)
	Itraconazole	3 (3.5%)	3 (3.5%)	0 (0%)	20 (23.3%)	27 (31.4%)	33 (38.4%)
	Caspofungin	2 (13.3%)	0 (0%)	0 (0%)	1 (6.7%)	4 (26.7%)	8 (53.3%)
	Micafungin	1 (5.9%)	2 (11.8%)	0 (0%)	1 (5.9%)	10 (58.8%)	3 (17.6%)
	Amphotericin B	13 (15.9%)	9 (11.0%)	0 (0%)	7 (8.5%)	12 (14.6%)	41 (50.0%)
	Flucytosine	1 (33.3%)	1 (33.3%)	0 (0%)	0 (0%)	0 (0%)	1 (33.3%)
Torsade de pointes/QT prolongation	Isavuconazole	2 (50.0%)	0 (0%)	0 (0%)	1 (25.0%)	1 (25.0%)	0 (0%)
	Posaconazole	9 (11.4%)	19 (24.1%)	0 (0%)	14 (17.7%)	27 (34.2%)	10 (12.7%)
	Voriconazole	18 (15.0%)	23 (19.2%)	0 (0%)	22 (18.3%)	30 (25.0%)	27 (22.5%)
	Fluconazole	14 (4.0%)	83 (24.0%)	0 (0%)	63 (18.2%)	84 (24.3%)	100 (28.9%)
	Itraconazole	6 (7.7%)	8 (10.3%)	0 (0%)	8 (10.3%)	35 (44.9%)	21 (26.9%)
	Caspofungin	0 (0%)	0 (0%)	0 (0%)	6 (35.3%)	4 (23.5%)	7 (41.2%)
	Micafungin	0 (0%)	1 (14.3%)	0 (0%)	0 (0%)	5 (71.4%)	1 (14.3%)
	Amphotericin B	4 (8.7%)	7 (15.2%)	0 (0%)	5 (10.9%)	11 (23.9%)	19 (41.3%)
	Flucytosine	0 (0%)	1 (50.0%)	0 (0%)	0 (0%)	0 (0%)	1 (50.0%)

## 4 Discussion

Systemic antifungal agents remain indispensable in the treatment and prevention of invasive fungal diseases; however, their real-world cardiac safety profiles have not been fully elucidated (Tisdale et al., 2020). The FAERS provides a valuable supplementary perspective for identifying “disproportionality signals”, which can be used to detect potential safety concerns warranting further epidemiologic and mechanistic validation; this paradigm has been widely adopted in recent pharmacovigilance studies across multiple therapeutic areas (Buchanan and Li, 2023; Khouri et al., 2021; Tan et al., 2024).

This study, based on FAERS data, focused on nine systemic antifungal agents: five triazoles (isavuconazole, posaconazole, voriconazole, fluconazole, itraconazole); two echinocandins (caspofungin, micafungin); one polyene (amphotericin B); and one pyrimidine analogue (flucytosine). Four disproportionality analysis methods were employed concurrently for cross-validation to mitigate false-positive or false-negative results associated with any single algorithm, thereby enhancing the robustness of the findings. To reduce study bias, SMQs were utilized instead of single PTs, and four arrhythmia-related SMQs were evaluated.

Overall, the ranking of antifungal agents by the number of SMQs showing positive signals was as follows: Itraconazole (4), Fluconazole (3), Posaconazole (3), Voriconazole (2), Caspofungin (1), Amphotericin B (1), Flucytosine (0), Isavuconazole (0), and Micafungin (0).

Comparative analysis revealed that the SMQ for “QT/TdP” exhibited the highest heterogeneity and the most prominent overall signal strength, with RORs ranging from 1.40 to 14.55. Among the triazole antifungals, fluconazole, posaconazole, itraconazole, and voriconazole all exhibited positive signal. In contrast, only isavuconazole showed a negative signal (ROR: 1.40, 95% CI: 0.53–3.74). This pattern aligns in direction with previously reported differences in the pharmacological profiles of triazoles and the consensus on the need for enhanced QT monitoring for certain agents (Cao et al., 2026; Ellsworth and Ostrosky-Zeichner, 2020; Khatib et al., 2021; Tisdale et al., 2020; Yu and Liao, 2022). Although the reason may be related to the least number of reports for isavuconazole (Tian et al., 2025), the most likely reason is its potential advantages, including good oral bioavailability (∼100%), lack of QTc interval prolongation, predictable pharmacokinetics, less complex drug interaction characteristics, and improved tolerability (Lewis et al., 2022; Candel et al., 2025). Among the echinocandins, positive signals were observed for caspofungin (ROR: 3.00, 95% CI: 1.89–4.76) and micafungin (ROR: 2.44, 95% CI: 1.35–4.42). Amphotericin B also exhibited a positive signal (ROR: 3.64, 95% CI: 2.83–4.69). Studies suggest that amphotericin B-associated electrolyte disturbances (e.g., hypokalemia, hypomagnesemia) may increase arrhythmogenic risk by promoting repolarization abnormalities, which is consistent with clinical management recommendations. Although flucytosine, a pyrimidine analog, had a ROR value > 1 (ROR 4.96, 95% CI: 1.24–19.92), it was considered a negative signal due to only two reported cases; this wide confidence interval indicates substantial uncertainty in “signal strength” under sparse counts and warrants cautious interpretation (Khouri et al., 2021).

Regarding the “Bradyarrhythmias” SMQ, positive signals were also predominantly concentrated among triazoles. The RORs for posaconazole, fluconazole, itraconazole, and voriconazole were 5.53, 5.53, 5.10, and 2.53, respectively. Isavuconazole again did not show a positive signal (ROR: 0.86, 95% CI: 0.32–2.28). Among non-triazole agents, only caspofungin presented a positive signal (ROR: 2.44, 95% CI: 1.63–3.64). The results are consistent with previously reported adverse reaction cases and database analysis (Uludağ et al., 2013; Panos et al., 2016; Cao et al., 2026).

For the SMQ of “Tachyarrhythmias”, the overall signal strength was generally lower than that for QT/TdP. However, several drugs still showed clinically significant disproportionality. The agents presenting positive signals were all triazoles: fluconazole (ROR: 3.53), posaconazole (ROR: 2.59), and itraconazole (ROR: 2.48). Although previous studies have reported that isavuconazole and voriconazole may induce tachycardia, they were not identified as positive signals in the present study may be related to factors such as insufficient report numbers and study bias (Dewan et al., 2017; Keirns et al., 2017).

It is noteworthy that the SMQ for “Cardiac arrhythmia terms, nonspecific” demonstrated relatively poor discriminatory power. Most drugs had RORs close to 1 with wide confidence intervals, with only itraconazole showing a positive signal. This result methodologically underscores the importance of SMQ selection; SMQs more closely aligned with specific clinical phenotypes (e.g., QT/TdP or bradyarrhythmias) are more likely to yield interpretable and comparable signals. In contrast, non-specific SMQs may dilute or confound events of different phenotypes, thereby reducing discriminatory power.

Figure 4 illustrates the differences among various antifungal agents under four SMQs. It is evident that the ranking and intensity of signals for different SMQs associated with antifungal drugs are not consistent. This suggests that the risk may not stem from a single arrhythmogenic mechanism but rather from the synthesis of multiple factors, including direct electrophysiological effects, drug-drug interactions, inhibition of hERG channel, changes in serum potassium levels, and patient susceptibility, etc (Xie et al., 2025; Syridou et al., 2026; Javandoust et al., 2024).

Sensitivity analysis revealed that the arrhythmia-related signals were largely consistent between population receiving and not receiving chemotherapy (Figure 5), and the SMQ for “QT/TdP” was more prominent in the chemotherapy population. This result suggests that in the context of chemotherapy, the increased burden of concomitant medications, electrolyte imbalances, and increased susceptibility to underlying cardiac events may collectively contribute to an increased likelihood of arrhythmia.

In addition to signal strength detection, Table 4 summarizes patient outcomes from the reports. For QT/TdP, death and life-threatening outcomes were recurrently reported for multiple drugs and were more prominent among triazoles with high signal strength. Concurrently, a high proportion of “unknown” outcomes indicates that missing outcome documentation remains common in spontaneous reporting systems. For “Tachyarrhythmias”, hospitalization/prolonged hospitalization was more frequently reported than death or life-threatening outcomes. This distribution of outcomes aligns with clinical consensus that drug-related rhythm abnormalities, particularly QT/TdP, can deteriorate rapidly and necessitate monitoring and risk management (Zeppenfeld et al., 2022).

Overall, the findings of this study offer several implications for clinical practice. First, fluconazole, posaconazole, and itraconazole showed strong positive signals for both QT/TdP and bradyarrhythmias, suggesting that these agents should be used cautiously when a suitable alternative exists, particularly in populations with pre-existing QT prolongation, electrolyte disturbances, cardiac disease, or a high risk of drug interactions (Lester et al., 2024; Liabeuf et al., 2025; Mehanna and Graaf, 2024; Nakano et al., 2025). Second, isavuconazole consistently showed negative signals across multiple cardiotoxicity-related SMQs in this analysis, indicating it may be a preferable alternative in specific clinical scenarios. This observation aligns with the direction of recent mechanistic and clinical evidence (Ellsworth and Ostrosky-Zeichner, 2020; Ergün et al., 2024; Neofytos et al., 2024). However, it may also be related to the relatively low number of adverse reaction reports, and more real-world evidence is needed to confirm this. Third, while non-triazole agents like amphotericin B and caspofungin exhibited generally milder signals, they are not “zero-risk”. Clinical vigilance remains necessary regarding electrolyte management (especially potassium and magnesium), infusion-related reactions, and concomitant medication risks to avoid the accumulation of factors predisposing to cardiotoxicity (Abdel-Hafez et al., 2022). In addition, FAERS-based analyses have suggested potential sex-specific reporting patterns for voriconazole AEs, supporting demographic-aware surveillance (Xu et al., 2025).

This study has several limitations. First, data from FAERS are derived from spontaneous reports, which are susceptible to under-reporting, missing information, and confounding factors such as indication and concomitant medications. Due to the limited number of complete reports, subgroup analyses of relevant influencing factors could not be performed, potentially introducing bias. Second, this study can only demonstrate an association between specific drugs and adverse reactions and cannot directly establish a causal relationship. Therefore, future confirmatory studies and mechanistic studies are warranted to clarify the impact of specific antifungal agents on cardiotoxicity (Boodman and Gupta, 2023). In addition, future work may incorporate standardized QT assessment frameworks (including regulatory guidance and model-informed approaches) to better judge the clinical significance of signals.

## 5 Conclusion

Based on FAERS database, this study employed four disproportionality analysis methods (ROR, PRR, BCPNN, and MGPS) to analyze the association between nine systemic antifungal agents and cardiotoxicity-related signals. Itraconazole, fluconazole, and posaconazole demonstrated stronger cardiotoxicity risks, whereas micafungin, flucytosine, and isavuconazole showed negative signals across all four SMQs. In clinical practice, individual patient risk should be comprehensively assessed to guide personalized drug selection, with reinforcement of electrolyte management, drug interaction review, and electrocardiographic monitoring.

## Data Availability

The original contributions presented in the study are included in the article/Supplementary Material, further inquiries can be directed to the corresponding author.

## References

[B1] Abdel-Hafez Y. Siaj H. Janajri M. Abu-Baker Y. Nazzal Z. Hamdan Z. (2022). Tolerability and epidemiology of nephrotoxicity associated with conventional amphotericin B therapy: a retrospective study in tertiary care centers in Palestine. BMC Nephrol. 23, 132. 10.1186/s12882-022-02770-2 35382766 PMC8982299

[B2] Boodman C. Gupta N. (2023). Schrödinger's cat paradox: bartonella serology cannot be used to speciate bartonella endocarditis. Open Forum Infect. Dis. 10 (8), ofad436. 10.1093/ofid/ofad436 37663087 PMC10468726

[B3] Buchanan J. M. Li M. (2023). Important considerations for signal detection and evaluation. Ther. Innov. Regul. Sci. 57, 865–874. 10.1007/s43441-023-00518-0 37067682 PMC10276783

[B4] Candel F. J. Matesanz M. Mensa J. Azanza J. R. (2025). Pharmacokinetic novelties of isavuconazole. Use in special situations. Rev. Iberoam. Micol. 42, 37–44. 10.1016/j.riam.2025.02.003 40240233

[B5] Cao D. Wei C. Yuan Y. Wu B. (2026). Cardiotoxicity associated with antifungal agents: a pharmacovigilance analysis of the FDA adverse event reporting system. Int. J. Clin. Pharmacol. Ther. 64, 103–114. 10.5414/CP204897 41378851

[B6] Chen J. Xu S. Yu W. Sun C. Zhang W. (2024). Evaluating cardiac disorders associated with triazole antifungal agents based on the US food and drug administration adverse event reporting system database. Front. Pharmacol. 15, 1255918. 10.3389/fphar.2024.1255918 38584605 PMC10997335

[B7] Dagher H. Hachem R. Chaftari A.-M. Jiang Y. Ali S. Deeba R. (2022). Real-world use of isavuconazole as primary therapy for invasive fungal infections in high-risk patients with hematologic malignancy or stem cell transplant. J. Fungi 8 (1), 74. 10.3390/jof8010074 PMC8779319 35050014

[B8] Denning D. W. (2024). Global incidence and mortality of severe fungal disease. Lancet Infect. Dis. 24, e428–e438. 10.1016/S1473-3099(23)00692-8 38224705

[B9] Dewan P. Gomber S. Arora V. (2017). Ventricular tachycardia: a rare side effect of voriconazole. Indian J. Pediatr. 84, 152–153. 10.1007/s12098-016-2217-9 27553662

[B10] Ellsworth M. Ostrosky-Zeichner L. (2020). Isavuconazole: Mechanism of Action, Clinical Efficacy, and Resistance. J. Fungi (Basele), 6(4), 324. 10.3390/jof6040324 33260353 PMC7712939

[B11] Ergün M. Jansen A. M. E. Hilbrands L. B. de Kort E. Kunst H. Reijers M. H. E. (2024). Isavuconazole as prophylaxis and therapy for invasive fungal diseases: a real-life observational study. J. Antimicrob. Chemother. 79, 1801–1810. 10.1093/jac/dkae139 38935893 PMC11290874

[B12] Fernandes M. A. Mota M. N. Faria N. T. Sá-Correia I. (2023). An evolved strain of the oleaginous yeast Rhodotorula toruloides, multi-tolerant to the major inhibitors present in lignocellulosic hydrolysates, exhibits an altered cell envelope. J. Fungi 9, 1073. 10.3390/jof9111073 PMC10672028 37998878

[B13] Fusaroli M. Salvo F. Begaud B. AlShammari T. M. Bate A. Battini V. (2024a). The reporting of a disproportionality analysis for drug safety signal detection using individual case safety reports in PharmacoVigilance (READUS-PV): development and statement. Drug Saf. 47, 575–584. 10.1007/s40264-024-01421-9 38713346 PMC11116242

[B14] Fusaroli M. Salvo F. Begaud B. AlShammari T. M. Bate A. Battini V. (2024b). The Reporting of A disproportionality analysis for drug safety signal detection using individual case safety reports in PharmacoVigilance (READUS-PV): explanation and elaboration. Drug Saf. 47, 585–599. 10.1007/s40264-024-01423-7 38713347 PMC11116264

[B15] Giunchi V. Fusaroli M. Hauben M. Raschi E Poluzzi E. (2023). Challenges and opportunities in accessing and analysing FAERS data: a call towards a collaborative approach. Drug Saf. 46, 921–926. 10.1007/s40264-023-01345-w 37651086

[B16] Hung E. Hauben M. Essex H. Bright C. Z. S. (2023). More extreme duplication in FDA adverse event reporting system detected by literature reference normalization and fuzzy string matching. Pharmacoepidemiol Drug Saf. 32, 387–391. 10.1002/pds.5555 36369928

[B17] Javandoust Gharehbagh F. Roshanzamiri S. Farjami M. Hatami F. Lotfollahi L. Kazeminia N. (2024). Liposomal amphotericin for secondary prophylaxis: a systematic review and meta-analysis. J. Oncol. Pharm. Pract. 30, 919–929. 10.1177/10781552241241317 38720564

[B18] Jiang M. Li H. Kong L. (2024). Data mining and safety analysis of dual orexin receptor antagonists (DORAs): a real-world pharmacovigilance study based on the FAERS database. Front. Pharmacol. 15, 1436405. 10.3389/fphar.2024.1436405 39166117 PMC11333359

[B19] Keirns J. Desai A. Kowalski D. Lademacher C. Mujais S. Parker B. (2017). QT interval shortening with isavuconazole: *in vitro* and *in vivo* effects on cardiac repolarization. Clin. Pharmacol. Ther. 101, 782–790. 10.1002/cpt.620 28074556 PMC5485736

[B20] Khatib R. Sabir F. R. N. Omari C. Pepper C. Tayebjee M. H. (2021). Managing drug-induced QT prolongation in clinical practice. Postgrad. Med. J. 97, 452–458. 10.1136/postgradmedj-2020-138661 33122341 PMC8237186

[B21] Khouri C. Nguyen T. Revol B. Lepelley M. Pariente A. Roustit M. (2021). Leveraging the variability of pharmacovigilance disproportionality analyses to improve signal detection performances. Front. Pharmacology 12, 668765. 10.3389/fphar.2021.668765 PMC8193489 34122089

[B22] Kriegl L. Egger M. Boyer J. Hoenig M Krause R. (2025). New treatment options for critically important WHO fungal priority pathogens. Clin. Microbiol. Infect. 31, 922–930. 10.1016/j.cmi.2024.03.006 38461942

[B23] Lester R. M. Engel C. van Haarst A. D. Paglialunga S. (2024). Should you run a dedicated TQT study? Sponsor and regulatory considerations on substitution pathways to assess QT liability. Clin. Pharmacol. Ther. 116, 42–51. 10.1002/cpt.3284 38698592

[B24] Lewis R. E. Wiederhold N. P. (2023). “Systemic antifungal agents,” in Diagnosis and Treatment of Fungal Infections. Springer, 125–147.

[B25] Lewis J. S. 2nd Wiederhold N. P. Hakki M. Thompson G. R. 3rd (2022). New perspectives on antimicrobial agents: isavuconazole. Antimicrob. Agents Chemother., 66, e0017722, 10.1128/aac.00177-22 35969068 PMC9487460

[B26] Li D. Chai S. Wang H. Dong J. Qin C. Du D. (2023). Drug-induced QT prolongation and torsade de pointes: a real-world pharmacovigilance study using the FDA Adverse Event Reporting System database. Front. Pharmacol. 14, 1259611. 10.3389/fphar.2023.1259611 38186652 PMC10771307

[B27] Li H. Jiang M. Pan X. Kong L. (2025). Data mining and safety analysis of voriconazole in patients with a hematological malignant tumor based on the FAERS database: differences between children and adults. Front. Pharmacol. 16, 1524702. 10.3389/fphar.2025.1524702 39925849 PMC11802493

[B28] Liabeuf S. Berdougo-Tritz J. Augey L. Laville S. M. Mbarek A. Deray G. (2025). QT-Prolonging medications: prevalence of use and associated risks in CKD. Nephrol. Dial. Transpl. 41, 614–624. 10.1093/ndt/gfaf196 PMC13037475 40996451

[B29] Mehanna M. P. van derGraaf H. (2024). Weaning off thorough QT studies. Clin. Pharmacol. Ther. 116, 11–13. 10.1002/cpt.3294 38879890

[B30] Nakano Y. Kotake K. Asai Y. Murata M. Uchiyama M. Shimono N. (2025). Adverse effects of posaconazole on adrenal steroid biosynthesis: an integrative approach using FAERS-based pharmacovigilance and systematic review with meta-analysis of randomised controlled trials. Int. J. Antimicrob. Agents 67, 107676. 10.1016/j.ijantimicag.2025.107676 41297713

[B31] Neofytos D. Pagliuca A. Houghton K. Broughton E. de Figueiredo Valente M. L. N. Jiang L. (2024). Effectiveness, safety, and patterns of real-world isavuconazole use in Europe (2015–2019). Infect. Dis. Ther. 13, 2527–2543. 10.1007/s40121-024-01064-4 39443403 PMC11582280

[B32] Ohyama K. Akiyama S. Iida M. Hori Y. (2023). Association of Torsade de Pointes and QT Prolongation With Antifungal Triazoles: analysis Using a Pharmacovigilance Database. In Vivo 37, 2719–2725. 10.21873/invivo.13382 37905641 PMC10621426

[B33] Panos G. Velissaris D. Karamouzos V. Matzaroglou C. Tylianakis M. (2016). Long QT syndrome leading to multiple cardiac arrests after posaconazole administration in an immune-compromised patient with sepsis: an unusual case report. Am. J. Case Rep. 17, 295–300. 10.12659/ajcr.896946 27125217 PMC4913753

[B34] Syridou G. Karagiannidou S. Papakonstantinou M. E. Manzana Peteinelli M. Giannakopoulos K. Papaevangelou V. (2026). Liposomal amphotericin B-induced cardiac arrhythmias in infantile visceral leishmaniasis: a case report. Pediatr. Infect. Dis. J. 45, e43–e46. 10.1097/INF.0000000000005015 41104916

[B35] Tan H. Yan X. Chen Y. Huang G. Luo L. Li W. (2024). A real-world pharmacovigilance study of drug-induced QT interval prolongation: analysis of spontaneous reports submitted to FAERS. Front. Cardiovasc. Med. 11, 1363382. 10.3389/fcvm.2024.1363382 38803662 PMC11128590

[B36] Thompson G. R. Chen S. C. A Alfouzan W. A. Izumikawa K Colombo A. L Maertens J (2024). A global perspective of the changing epidemiology of invasive fungal disease and real-world experience with the use of isavuconazole. Med. Mycol., 62, (9):myae083, 10.1093/mmy/myae083 39138063 PMC11382804

[B37] Tian Y. Jin M. Ning H. (2025). A post-marketing pharmacovigilance study of triazole antifungals: adverse event data mining and analysis based on the FDA adverse event reporting system database. Front. Pharmacol. 16, 1462510. 10.3389/fphar.2025.1462510 39917625 PMC11799232

[B38] Tisdale J. E. Chung M. K. Campbell K. B. Hammadah M. Joglar J. A. Leclerc J. (2020). Drug-induced arrhythmias: a scientific statement from the American heart association. Circulation 142, e214–e233. 10.1161/CIR.0000000000000905 32929996

[B39] Uludağ D. Ozdemir N. Tüysüz G. Eroğlu A. G. Celkan T. (2013). Voriconazole induced bradycardia. Pediatr. Hematol. Oncol. 30, 674–676. 10.3109/08880018.2013.775616 23484777

[B40] Ünal Yüksekgönül A. Karagöz İ. E. T. Karagöz T. (2021). Fluconazole-associated QT interval prolongation and Torsades de Pointes in a paediatric patient. Cardiol. Young 31, 2035–2037. 10.1017/S1047951121001992 34024302

[B41] Xie P. Lu L. Tian Y. Jia R. Bai X. T. P. (2025). Cardiac arrhythmias of BCR-ABL inhibitors with or without triazole antifungal agents: a real-world pharmacovigilance study based on the food and drug administration adverse event reporting system database. SAGE Open Med. 13, 20503121251328762. 10.1177/20503121251328762 40143928 PMC11938897

[B42] Xu Q. Cheng H. Sun X. Zhao J. Chen Y. Ji L. (2025). A real-world pharmacovigilance study of FDA adverse event reporting system (FAERS) events for gender of voriconazole drugs. Drug Res. (Stuttg) 75, 218–224. 10.1055/a-2575-1530 40294598 PMC12227254

[B43] Yu Z. Liao X. (2022). Torsade de Pointes/QT Prolongation Associated with Antifungal Triazoles: a Pharmacovigilance Study Based on the U.S. FDA Adverse Event Reporting System(FAERS). J. Pharm. Pharm. Sci. 25, 237–243. 10.18433/jpps32867 35790147

[B44] Zeppenfeld K. Tfelt-Hansen J. de Riva M. Winkel B. G. Behr E. R. Blom N. A. (2022). 2022 ESC guidelines for the management of patients with ventricular arrhythmias and the prevention of sudden cardiac death. Eur. Heart J. 43, 3997–4126. 10.1093/eurheartj/ehac262 36017572

[B45] Zhang Z. Bills G. F An Z. (2023). Advances in the treatment of invasive fungal disease. PLoS Pathog. 19, e1011322. 10.1371/journal.ppat.1011322 37141208 PMC10159104

