# Supplementary material for: Comparative risk of arrhythmias associated with systemic antifungal agents: a disproportionality analysis of the FAERS database
Source: Front Pharmacol. 2026 May 26;17:1804602. doi: 10.3389/fphar.2026.1804602 (PMC13247426; doi:10.3389/fphar.2026.1804602)
Supplement: Supplementary file 1 [file Table1.docx]

**Supplementary Table 1.** The specific Preferred Terms (PTs) included in each SMQ.

| **SMQs** | **PTs** | **Code** |
| --- | --- | --- |
| **Cardiac arrhythmia terms, nonspecific** |  |  |
|  | ARRHYTHMIA | 10003119 |
|  | HEART ALTERNATION | 10058155 |
|  | HEART RATE IRREGULAR | 10019304 |
|  | HOLIDAY HEART SYNDROME | 10083709 |
|  | PACEMAKER GENERATED ARRHYTHMIA | 10053486 |
|  | PACEMAKER SYNDROME | 10051994 |
|  | PAROXYSMAL ARRHYTHMIA | 10050106 |
|  | PULSELESS ELECTRICAL ACTIVITY | 10058151 |
|  | REPERFUSION ARRHYTHMIA | 10058156 |
|  | WITHDRAWAL ARRHYTHMIA | 10047997 |
| **Bradyarrhythmias** |  |  |
|  | BRADYARRHYTHMIA | 10049765 |
|  | ICTAL BRADYCARDIA SYNDROME | 10088979 |
|  | SUPRAVENTRICULAR BRADYCARDIA | 10090651 |
|  | VENTRICULAR ASYSTOLE | 10047284 |
|  | ACCESSORY CARDIAC PATHWAY | 10067618 |
|  | ADAMS-STOKES SYNDROME | 10001115 |
|  | AGONAL RHYTHM | 10054015 |
|  | ATRIAL CONDUCTION TIME PROLONGATION | 10064191 |
|  | ATRIAL ESCAPE RHYTHM | 10085756 |
|  | ATRIAL STANDSTILL | 10087237 |
|  | ATRIOVENTRICULAR BLOCK | 10003671 |
|  | ATRIOVENTRICULAR BLOCK COMPLETE | 10003673 |
|  | ATRIOVENTRICULAR BLOCK FIRST DEGREE | 10003674 |
|  | ATRIOVENTRICULAR BLOCK SECOND DEGREE | 10003677 |
|  | ATRIOVENTRICULAR CONDUCTION TIME SHORTENED | 10068180 |
|  | ATRIOVENTRICULAR DISSOCIATION | 10069571 |
|  | ATRIOVENTRICULAR NODE DYSFUNCTION | 10084085 |
|  | BIFASCICULAR BLOCK | 10057393 |
|  | BRASH SYNDROME | 10084073 |
|  | BRUGADA SYNDROME | 10059027 |
|  | BUNDLE BRANCH BLOCK | 10006578 |
|  | BUNDLE BRANCH BLOCK BILATERAL | 10006579 |
|  | BUNDLE BRANCH BLOCK LEFT | 10006580 |
|  | BUNDLE BRANCH BLOCK RIGHT | 10006582 |
|  | CONDUCTION DISORDER | 10010276 |
|  | DEFECT CONDUCTION INTRAVENTRICULAR | 10012118 |
|  | ECTOPIC ATRIAL RHYTHM | 10088339 |
|  | ELECTROCARDIOGRAM DELTA WAVES ABNORMAL | 10014372 |
|  | ELECTROCARDIOGRAM PR PROLONGATION | 10053657 |
|  | ELECTROCARDIOGRAM PR SHORTENED | 10014374 |
|  | ELECTROCARDIOGRAM QRS COMPLEX PROLONGED | 10014380 |
|  | ELECTROCARDIOGRAM QT PROLONGED | 10014387 |
|  | ELECTROCARDIOGRAM REPOLARISATION ABNORMALITY | 10052464 |
|  | FASCICULAR BLOCK | 10086740 |
|  | LENEGRE'S DISEASE | 10071710 |
|  | LONG QT SYNDROME | 10024803 |
|  | PAROXYSMAL ATRIOVENTRICULAR BLOCK | 10077503 |
|  | SINOATRIAL BLOCK | 10040736 |
|  | TRIFASCICULAR BLOCK | 10044644 |
|  | VENTRICULAR DYSSYNCHRONY | 10071186 |
|  | WOLFF-PARKINSON-WHITE SYNDROME | 10048015 |
|  | ATRIAL ESCAPE RHYTHM | 10085756 |
|  | ECTOPIC ATRIAL RHYTHM | 10088339 |
|  | NODAL ARRHYTHMIA | 10029458 |
|  | NODAL RHYTHM | 10029470 |
|  | SINUS ARREST | 10040738 |
|  | SINUS ARRHYTHMIA | 10040739 |
|  | SINUS BRADYCARDIA | 10040741 |
|  | SINUS NODE DYSFUNCTION | 10075889 |
|  | WANDERING PACEMAKER | 10047818 |
| **Tachyarrhythmias** |  |  |
|  | ARRHYTHMIA SUPRAVENTRICULAR | 10003130 |
|  | ATRIAL FIBRILLATION | 10003658 |
|  | ATRIAL FLUTTER | 10003662 |
|  | ATRIAL PARASYSTOLE | 10071666 |
|  | ATRIAL TACHYCARDIA | 10003668 |
|  | CONGENITAL SUPRAVENTRICULAR TACHYCARDIA | 10082343 |
|  | FAMILIAL ATRIAL FIBRILLATION | 10088317 |
|  | FREDERICK'S SYNDROME | 10082089 |
|  | INAPPROPRIATE SINUS TACHYCARDIA | 10090513 |
|  | JUNCTIONAL ECTOPIC TACHYCARDIA | 10074640 |
|  | SINUS TACHYCARDIA | 10040752 |
|  | SUPRAVENTRICULAR EXTRASYSTOLES | 10042602 |
|  | SUPRAVENTRICULAR TACHYARRHYTHMIA | 10065342 |
|  | SUPRAVENTRICULAR TACHYCARDIA | 10042604 |
|  | ANOMALOUS ATRIOVENTRICULAR EXCITATION | 10002611 |
|  | ARRHYTHMIA INDUCED CARDIOMYOPATHY | 10091289 |
|  | CARDIAC FIBRILLATION | 10061592 |
|  | CARDIAC FLUTTER | 10052840 |
|  | EXTRASYSTOLES | 10015856 |
|  | TACHYARRHYTHMIA | 10049447 |
|  | ACCELERATED IDIOVENTRICULAR RHYTHM | 10049003 |
|  | ARRHYTHMIC STORM | 10067339 |
|  | CARDIAC FIBRILLATION | 10061592 |
|  | EARLY REPOLARISATION SYNDROME | 10086230 |
|  | PARASYSTOLE | 10033929 |
|  | RHYTHM IDIOVENTRICULAR | 10039111 |
|  | TORSADE DE POINTES | 10044066 |
|  | VENTRICULAR ARRHYTHMIA | 10047281 |
|  | VENTRICULAR EXTRASYSTOLES | 10047289 |
|  | VENTRICULAR FIBRILLATION | 10047290 |
|  | VENTRICULAR FLUTTER | 10047294 |
|  | VENTRICULAR PARASYSTOLE | 10058184 |
|  | VENTRICULAR PRE-EXCITATION | 10049761 |
|  | VENTRICULAR TACHYARRHYTHMIA | 10065341 |
|  | VENTRICULAR TACHYCARDIA | 10047302 |
| **Torsade de pointes/QT prolongation** |  |  |
|  | Electrocardiogram QT interval abnormal | 10063748 |
|  | Electrocardiogram QT prolonged | 10014387 |
|  | Long QT syndrome | 10024803 |
|  | Long QT syndrome congenital | 10057926 |
|  | Torsade de pointes | 10044066 |
|  | Ventricular tachycardia | 10047302 |

**Supplementary Table 2.** Signal intensity of arrhythmogenic toxicity caused by systemic antifungal drugs in patients receiving chemotherapy.

| **SMQs** | **Drug** | **Frequency** | **ROR(95%Cl)** | **PRR(χ2)** | **EBGM(EBGM05)** | **IC(IC025)** | **Positive signal** |
| --- | --- | --- | --- | --- | --- | --- | --- |
| **Cardiac arrhythmia terms, nonspecific** | Isavuconazole | 0 | NA | NA | NA | NA | No |
|  | Posaconazole | 5 | 1.59 ( 0.66 - 3.83 ) | 1.59 ( 1.1 ) | 1.59 ( 0.66 ) | 0.67 ( -0.65 ) | No |
|  | Voriconazole | 5 | 0.83 ( 0.35 - 2 ) | 0.83 ( 0.17 ) | 0.83 ( 0.35 ) | -0.26 ( -1.4 ) | No |
|  | Fluconazole | 5 | 1.91 ( 0.8 - 4.6 ) | 1.91 ( 2.17 ) | 1.91 ( 0.79 ) | 0.93 ( -0.45 ) | No |
|  | Itraconazole | 28 | 2.27 ( 1.86 - 2.78 ) | 2.27 ( 66.71 ) | 2.27 ( 1.85 ) | 1.18 ( 0.87 ) | Yes |
|  | Caspofungin | 1 | 1.48 ( 0.21 - 10.5 ) | 1.48 ( 0.15 ) | 1.48 ( 0.21 ) | 0.56 ( -1.79 ) | No |
|  | Micafungin | 1 | 1.09 ( 0.15 - 7.74 ) | 1.09 ( 0.01 ) | 1.09 ( 0.15 ) | 0.12 ( -1.98 ) | No |
|  | Amphotericin B | 1 | 0.62 ( 0.09 - 4.41 ) | 0.62 ( 0.23 ) | 0.62 ( 0.09 ) | -0.69 ( -2.43 ) | No |
|  | Flucytosine | 0 | NA | NA | NA | NA | No |
| **Bradyarrhythmias** | Isavuconazole | 3 | 4.68 ( 1.5 - 14.58 ) | 4.65 ( 8.62 ) | 4.65 ( 1.49 ) | 2.22 ( -0.17 ) | No |
|  | Posaconazole | 18 | 4.95 ( 3.22 - 7.61 ) | 4.92 ( 65.66 ) | 4.92 ( 3.2 ) | 2.3 ( 1.44 ) | Yes |
|  | Voriconazole | 20 | 2.96 ( 1.98 - 4.41 ) | 2.95 ( 30.89 ) | 2.95 ( 1.97 ) | 1.56 ( 0.87 ) | No |
|  | Fluconazole | 14 | 4.53 ( 2.77 - 7.41 ) | 4.5 ( 43.66 ) | 4.5 ( 2.75 ) | 2.17 ( 1.2 ) | Yes |
|  | Itraconazole | 80 | 5.21 ( 4.65 - 5.85 ) | 5.18 ( 980.64 ) | 5.17 ( 4.61 ) | 2.37 ( 2.18 ) | Yes |
|  | Caspofungin | 6 | 8.81 ( 4.38 - 17.7 ) | 8.7 ( 54.58 ) | 8.7 ( 4.33 ) | 3.12 ( 1.26 ) | Yes |
|  | Micafungin | 1 | 4.84 ( 2.17 - 10.82 ) | 4.81 ( 18.16 ) | 4.81 ( 2.16 ) | 2.27 ( 0.54 ) | Yes |
|  | Amphotericin B | 6 | 2.75 ( 1.24 - 6.14 ) | 2.75 ( 6.67 ) | 2.75 ( 1.23 ) | 1.46 ( 0.04 ) | No |
|  | Flucytosine | 0 | NA | NA | NA | NA | No |
| **Tachyarrhythmias** | Isavuconazole | 1 | 0.79 ( 0.11 - 5.62 ) | 0.79 ( 0.06 ) | 0.79 ( 0.11 ) | -0.34 ( -2.23 ) | No |
|  | Posaconazole | 14 | 1.91 ( 1.17 - 3.13 ) | 1.91 ( 6.94 ) | 1.91 ( 1.17 ) | 0.93 ( 0.15 ) | No |
|  | Voriconazole | 39 | 2.83 ( 2.11 - 3.79 ) | 2.81 ( 52.73 ) | 2.81 ( 2.1 ) | 1.49 ( 1.01 ) | Yes |
|  | Fluconazole | 29 | 4.49 ( 3.15 - 6.41 ) | 4.44 ( 82.96 ) | 4.44 ( 3.12 ) | 2.15 ( 1.49 ) | Yes |
|  | Itraconazole | 75 | 3.15 ( 2.83 - 3.5 ) | 3.12 ( 499.14 ) | 3.12 ( 2.81 ) | 1.64 ( 1.48 ) | Yes |
|  | Caspofungin | 0 | NA | NA | NA | NA | No |
|  | Micafungin | 5 | 2.05 ( 0.85 - 4.94 ) | 2.04 ( 2.67 ) | 2.04 ( 0.85 ) | 1.03 ( -0.38 ) | No |
|  | Amphotericin B | 9 | 2.11 ( 1.09 - 4.06 ) | 2.1 ( 5.19 ) | 2.1 ( 1.09 ) | 1.07 ( 0 ) | No |
|  | Flucytosine | 0 | NA | NA | NA | NA | No |
| **Torsade de pointes/QT prolongation** | Isavuconazole | 2 | 7.62 ( 2.45 - 23.72 ) | 7.57 ( 17.11 ) | 7.57 ( 2.43 ) | 2.92 ( 0.07 ) | Yes |
|  | Posaconazole | 15 | 6.9 ( 4.34 - 10.97 ) | 6.86 ( 90.1 ) | 6.85 ( 4.31 ) | 2.78 ( 1.72 ) | Yes |
|  | Voriconazole | 35 | 7.03 ( 5.04 - 9.8 ) | 6.99 ( 179.64 ) | 6.98 ( 5.01 ) | 2.8 ( 2.1 ) | Yes |
|  | Fluconazole | 32 | 14.85 ( 10.48 - 21.06 ) | 14.65 ( 407.11 ) | 14.64 ( 10.33 ) | 3.87 ( 2.87 ) | Yes |
|  | Itraconazole | 77 | 12.69 ( 11.54 - 13.95 ) | 12.54 ( 4567.66 ) | 12.48 ( 11.35 ) | 3.64 ( 3.46 ) | Yes |
|  | Caspofungin | 7 | 12.52 ( 5.94 - 26.38 ) | 12.37 ( 73.25 ) | 12.37 ( 5.87 ) | 3.63 ( 1.32 ) | Yes |
|  | Micafungin | 2 | 6.56 ( 2.72 - 15.81 ) | 6.52 ( 23.4 ) | 6.52 ( 2.71 ) | 2.71 ( 0.58 ) | Yes |
|  | Amphotericin B | 5 | 3.73 ( 1.55 - 8.98 ) | 3.72 ( 9.96 ) | 3.72 ( 1.55 ) | 1.9 ( 0.17 ) | No |
|  | Flucytosine | 0 | NA | NA | NA | NA | No |

**Supplementary Table 3.** Signal intensity of arrhythmogenic toxicity caused by systemic antifungal drugs in patients receiving non-chemotherapy.

| **SMQs** | **Drug** | **Frequency** | **ROR(95%Cl)** | **PRR(χ2)** | **EBGM(EBGM05)** | **IC(IC025)** | **Positive signal** |
| --- | --- | --- | --- | --- | --- | --- | --- |
| **Cardiac arrhythmia terms, nonspecific** | Isavuconazole | 2 | 0.54 ( 0.14 - 2.17 ) | 0.54 ( 0.77 ) | 0.54 ( 0.14 ) | -0.88 ( -2.31 ) | No |
|  | Posaconazole | 10 | 0.9 ( 0.49 - 1.68 ) | 0.9 ( 0.1 ) | 0.9 ( 0.49 ) | -0.14 ( -1 ) | No |
|  | Voriconazole | 49 | 1 ( 0.75 - 1.32 ) | 1 ( 0 ) | 1 ( 0.75 ) | -0.01 ( -0.41 ) | No |
|  | Fluconazole | 105 | 2.45 ( 2.03 - 2.97 ) | 2.45 ( 90.07 ) | 2.45 ( 2.02 ) | 1.29 ( 0.99 ) | Yes |
|  | Itraconazole | 15 | 3.06 ( 2.3 - 4.06 ) | 3.05 ( 66.31 ) | 3.05 ( 2.3 ) | 1.61 ( 1.14 ) | Yes |
|  | Caspofungin | 8 | 1.05 ( 0.53 - 2.1 ) | 1.05 ( 0.02 ) | 1.05 ( 0.53 ) | 0.07 ( -0.9 ) | No |
|  | Micafungin | 7 | 3.06 ( 2.3 - 4.06 ) | 3.05 ( 66.31 ) | 3.05 ( 2.3 ) | 1.61 ( 1.14 ) | Yes |
|  | Amphotericin B | 31 | 1.41 ( 0.99 - 1.99 ) | 1.41 ( 3.77 ) | 1.41 ( 0.99 ) | 0.49 ( -0.03 ) | No |
|  | Flucytosine | 0 | NA | NA | NA | NA | No |
| **Bradyarrhythmias** | Isavuconazole | 3 | 0.2 ( 0.03 - 1.42 ) | 0.2 ( 3.21 ) | 0.2 ( 0.03 ) | -2.32 ( -3.63 ) | No |
|  | Posaconazole | 76 | 5.98 ( 4.85 - 7.37 ) | 5.93 ( 365.23 ) | 5.93 ( 4.81 ) | 2.57 ( 2.18 ) | Yes |
|  | Voriconazole | 137 | 2.54 ( 2.18 - 2.95 ) | 2.53 ( 156.76 ) | 2.53 ( 2.18 ) | 1.34 ( 1.1 ) | Yes |
|  | Fluconazole | 278 | 5.21 ( 4.65 - 5.84 ) | 5.18 ( 1013.89 ) | 5.17 ( 4.61 ) | 2.37 ( 2.18 ) | Yes |
|  | Itraconazole | 21 | 4.48 ( 3.66 - 5.48 ) | 4.45 ( 254.39 ) | 4.45 ( 3.63 ) | 2.15 ( 1.81 ) | Yes |
|  | Caspofungin | 18 | 2.33 ( 1.56 - 3.48 ) | 2.32 ( 18.13 ) | 2.32 ( 1.56 ) | 1.22 ( 0.56 ) | No |
|  | Micafungin | 10 | 4.48 ( 3.66 - 5.48 ) | 4.45 ( 254.39 ) | 4.45 ( 3.63 ) | 2.15 ( 1.81 ) | Yes |
|  | Amphotericin B | 39 | 1.56 ( 1.17 - 2.06 ) | 1.55 ( 9.49 ) | 1.55 ( 1.17 ) | 0.64 ( 0.21 ) | No |
|  | Flucytosine | 0 | NA | NA | NA | NA | No |
| **Tachyarrhythmias** | Isavuconazole | 8 | 1.02 ( 0.55 - 1.89 ) | 1.02 ( 0 ) | 1.02 ( 0.55 ) | 0.03 ( -0.85 ) | No |
|  | Posaconazole | 73 | 3.07 ( 2.5 - 3.78 ) | 3.05 ( 124.64 ) | 3.05 ( 2.48 ) | 1.61 ( 1.27 ) | Yes |
|  | Voriconazole | 144 | 1.24 ( 1.06 - 1.44 ) | 1.24 ( 7.28 ) | 1.24 ( 1.06 ) | 0.3 ( 0.08 ) | No |
|  | Fluconazole | 339 | 3.12 ( 2.81 - 3.46 ) | 3.1 ( 503.91 ) | 3.1 ( 2.79 ) | 1.63 ( 1.47 ) | Yes |
|  | Itraconazole | 22 | 2.42 ( 1.99 - 2.94 ) | 2.41 ( 83.46 ) | 2.41 ( 1.98 ) | 1.27 ( 0.96 ) | Yes |
|  | Caspofungin | 15 | 0.84 ( 0.52 - 1.35 ) | 0.84 ( 0.53 ) | 0.84 ( 0.52 ) | -0.25 ( -0.92 ) | No |
|  | Micafungin | 5 | 2.05 ( 0.85 - 4.94 ) | 2.04 ( 2.67 ) | 2.04 ( 0.85 ) | 1.03 ( -0.38 ) | No |
|  | Amphotericin B | 91 | 1.84 ( 1.52 - 2.21 ) | 1.83 ( 41.93 ) | 1.83 ( 1.52 ) | 0.87 ( 0.59 ) | No |
|  | Flucytosine | 3 | 1.92 ( 0.62 - 5.97 ) | 1.91 ( 1.31 ) | 1.91 ( 0.61 ) | 0.94 ( -0.81 ) | No |
| **Torsade de pointes/QT prolongation** | Isavuconazole | 2 | 0.65 ( 0.16 - 2.6 ) | 0.65 ( 0.38 ) | 0.65 ( 0.16 ) | -0.62 ( -2.11 ) | No |
|  | Posaconazole | 87 | 11.28 ( 9.29 - 13.7 ) | 11.17 ( 953.21 ) | 11.15 ( 9.18 ) | 3.48 ( 3.06 ) | Yes |
|  | Voriconazole | 137 | 3.35 ( 2.83 - 3.96 ) | 3.34 ( 224.35 ) | 3.34 ( 2.82 ) | 1.74 ( 1.47 ) | Yes |
|  | Fluconazole | 1 | 2.04 ( 0.29 - 14.49 ) | 2.04 ( 0.53 ) | 2.04 ( 0.29 ) | 1.03 ( -1.62 ) | No |
|  | Itraconazole | 13 | 5.82 ( 4.64 - 7.29 ) | 5.79 ( 301.22 ) | 5.79 ( 4.62 ) | 2.53 ( 2.12 ) | Yes |
|  | Caspofungin | 11 | 2.84 ( 1.79 - 4.51 ) | 2.83 ( 21.38 ) | 2.83 ( 1.78 ) | 1.5 ( 0.71 ) | No |
|  | Micafungin | 9 | 5.82 ( 4.64 - 7.29 ) | 5.79 ( 301.22 ) | 5.79 ( 4.62 ) | 2.53 ( 2.12 ) | Yes |
|  | Amphotericin B | 56 | 3.54 ( 2.78 - 4.5 ) | 3.53 ( 121.43 ) | 3.53 ( 2.77 ) | 1.82 ( 1.41 ) | Yes |
|  | Flucytosine | 1 | 2.04 ( 0.29 - 14.49 ) | 2.04 ( 0.53 ) | 2.04 ( 0.29 ) | 1.03 ( -1.62 ) | No |
